# In Vitro Toxicity Screening of Fifty Complex Mixtures in HepG2 Cells

**DOI:** 10.3390/toxics12020126

**Published:** 2024-02-02

**Authors:** Sunmi Kim, Kyounghee Kang, Haena Kim, Myungwon Seo

**Affiliations:** 1 Chemical Analysis Center, Korea Institute of Chemical Technology (KRICT), Daejeon 34114, Republic of Korea; coolcool@krict.re.kr (K.K.); hnkim@krict.re.kr (H.K.); mwseo@krict.re.kr (M.S.); 2 Department of Chemistry, Chungnam National University, Daejeon 34134, Republic of Korea

**Keywords:** complex mixture, cytotoxicity, mixture toxicity prediction, HepG2, biomonitoring

## Abstract

To develop the risk prediction technology for mixture toxicity, a reliable and extensive dataset of experimental results is required. However, most published literature only provides data on combinations containing two or three substances, resulting in a limited dataset for predicting the toxicity of complex mixtures. Complex mixtures may have different mode of actions (MoAs) due to their varied composition, posing difficulty in the prediction using conventional toxicity prediction models, such as the concentration addition (CA) and independent action (IA) models. The aim of this study was to generate an experimental dataset comprising complex mixtures. To identify the target complex mixtures, we referred to the findings of the HBM4EU project. We identified three groups of seven to ten components that were commonly detected together in human bodies, namely environmental phenols, perfluorinated compounds, and heavy metal compounds, assuming these chemicals to have different MoAs. In addition, a separate mixture was added consisting of seven organophosphate flame retardants (OPFRs), which may have similar chemical structures. All target substances were tested for cytotoxicity using HepG2 cell lines, and subsequently 50 different complex mixtures were randomly generated with equitoxic mixtures of EC10 levels. To determine the interaction effect, we calculated the model deviation ratio (MDR) by comparing the observed EC10 with the predicted EC10 from the CA model, then categorized three types of interactions: antagonism, additivity, and synergism. Dose–response curves and EC values were calculated for all complex mixtures. Out of 50 mixtures, none demonstrated synergism, while six mixtures exhibited an antagonistic effect. The remaining mixtures exhibited additivity with MDRs ranging from 0.50 to 1.34. Our experimental data have been formatted to and constructed for the database. They will be utilized for further research aimed at developing the combined CA/IA approaches to support mixture risk assessment.

## 1. Introduction

People are exposed to numerous chemical mixtures from consumer products, food, and the environment. These mixtures may have an impact on health. Therefore, to assess the mixture risk, we need to estimate the toxicity quantitatively. The European Union (EU) Biocidal Product Regulation (BPR) and the Korean Chemical Product Safety Act take into account the combined toxicity that can be caused by mixture components in consumer chemical products and biocidal products [1,2]. This is due to the fact that individual chemicals, even below their no-observed-effect levels, may provoke toxicity through toxicological interactions (e.g., additivity or synergism) among them in the living organisms [3,4]. The concept of additive toxicity has conventionally been applied without considering the possibility of synergistic interaction [5]. General models used to predict quantitative mixture toxicity values, such as EC10 or EC50, include the concentration addition (CA) and the independent action (IA) models [6,7]. The CA model, also called the dose addition model (DA), is suitable for mixtures consisting of substances with the same mode of action (MoA), and this is the general default assumption in most mixture risk assessment concepts.

The default use of the CA model is necessary and practical for regulatory applications because the biological MoAs are not known for all components, and synergistic interaction cases are rare [8]. However, in order to achieve a more comprehensive prediction of mixture risk, it is essential to develop quantitative models for predicting mixture toxicity. This is because conducting conventional toxicity tests is not feasible due to the extremely large number of possible mixture combinations, including different chemical types and concentrations. The in-depth mechanism of mixture toxicity is always complex, making it difficult to clearly understand the interaction effect of chemical mixtures on target species and assay conditions [9]. In response to this research need, several mixture toxicity prediction models have been developed, including the generalized concentration addition (GCA) model [10], the stepwise modelling combined CA and IA models [11,12], and the full logistic model (FLM) [13]. To support the mixture toxicity prediction, our research team has developed a web-based platform that conveniently estimates the mixture toxicity values based on various prediction models [14]. However, the computational prediction models still require improvement. To enhance their predictive power, a larger dataset of observed toxicity values of complex mixtures is necessary; however, such information on the toxicity of complex mixtures is still very limited.

A complex mixture is a combination of multiple components with different MoAs, often containing around 10 chemicals [11,15,16,17]. In 2004, a peer review workshop of the US EPA recognized the need to develop novel approaches for assessing the risk of complex mixtures and producing toxicity test results that take into account real-world exposure conditions rather than conventional simple mixtures [17]. In reality, we are exposed to the complex mixtures with various toxicity mechanisms. Large-scale human biomonitoring (HBM) projects, such as the US National Health and Nutrition Examination Survey (NHANES) and human biomonitoring for Europe (HBM4EU), have demonstrated significantly that exogenous chemicals and their metabolites are detected in blood, urine, and other human specimens [18,19]. However, a recent review of mixture toxicity data found that less than 20% of cases have tested a mixture including more than three components [3]. It is worth noting that the US EPA Peer Review Workshop report was published over 15 years ago.

The aim of our study is to provide a toxicity experimental dataset of complex mixtures under the same assay conditions. For this study, we selected major exogenous chemicals based on human biomonitoring data and randomly organized them into mixtures of seven to ten components. In complex mixtures, we ensured that there was a group with similar structures and functions and another group with various characteristics of toxicity, regarding the application of both the CA and IA models. To account for complex cases, we combined the chemicals randomly, and generated fifty different mixtures. Our study results can be used to develop and optimize the quantitative prediction models for mixture toxicity in future work.

## 2. Materials and Methods

### 2.1. Target Components and Mixtures

In order to generate the target complex mixtures, eighteen environmental chemicals were selected from the HBM4EU project data sample, along with seven additional OPFRs that might have similar structures and functions. For environmental chemicals, we referred to examples of common chemicals found in the HBM4EU project data sample. The Deliverable Report 15.3 of this project provided a data re-analysis approach and case study using simulated data of real-life exposure profiles in human biomonitoring [20]. Although this is based on the simulated data, we utilized it to generate the toxicity data without attributing any biological significance. In the report, 10 mixtures are presented which account for 65.8% of the variance of the original HBM dataset. We have selected the top 4 mixtures, which cover a total of 49% of the variance. Detailed chemical lists are available in the report, including brominated flame retardants, heavy metals, environmental phenols, phthalates, per- and polyfluoroalkyl substances (PFAS), and polychlorinated biphenyls (PCBs). In the list, we excluded low-toxic chemicals, such as phthalates from our pre-test. We also excluded persistent organic pollutants (POPs) due to regulations restricting their purchase above a certain concentration and quantity. From this process, we selected eighteen chemicals from the HBM4EU example dataset. For the next step, we selected the most frequently detected organophosphate flame retardants (OPFRs) from previous studies to consider the chemical group with similar structures and functions [21,22]. In our previous study, we identified seven OPFRs that showed significant cytotoxicity in HepG2 toxicity assay. Most of the combinations had no interaction effects in the mixture toxicity test [21].

We first selected ‘Mixture 2, 3, and 4’ of the HBM4EU report as the representative target mixtures, and then included all seven OPFRs for the next mixture. Subsequently, we randomly generated 46 mixtures to include 7 to 10 components, and they were made into EC10-level equitoxic mixtures. Table 1 shows the final target single components and representative four mixtures, while other mixtures are summarized in Table S1.

### 2.2. HepG2 Cell Line Culture and Cytotoxicity Assay

The majority of components in the complex mixtures are heavy metals and semi-volatile organic chemicals (SVOCs), which can be orally exposed to general population [23,24,25]. As an experimental setting, we selected the hepatocyte cell line HepG2, considering their circulation and general toxicity. The HepG2 cell line has been used extensively in toxicity studies, providing sufficient information on biological mechanisms and allowing for easy comparison with previous studies.

The HepG2 cell lines were obtained from CLS (Cell Lines Service, Eppelheim, Germany), and the cells were cultured in Dulbecco’s Modified Eagle’s Medium (DMEM, WelGENE Inc., Gyeongsan, Korea, LM001-05) supplemented with 10% fetal bovine serum (FBS, Atlas, Atlanta, GA, USA, FP-0500-A) and 1% penicillin-streptomycin (PE-ST, Gibco, Grand Island, NY, USA, 15070063) in 100 × 25 mm round culture dishes (Corning, Corning, NY, USA) at 37 °C and 5% CO_2_. For the cytotoxicity assay, the cells were dispensed at a concentration of 1.5 × 10^5^/mL in 96-well plates. After incubating for 24 h, the culture medium was replaced with solutions containing the target substances and then exposed for 48 h. All target chemicals were dispersed or diluted to a concentration of 1 mM in a dimethyl sulfoxide (DMSO, Hybri-Max™, ≥99.7%, Sigma-Aldrich, St. Louis, MO, USA, D2650). Sodium selenite, antimony (III) chloride, cobalt chloride, and nickel dichloride were dispersed with ultrasonication for 30 to 60 min using an ultrasonic probe and homogenizer. Then chemicals were treated in eight treatment groups with dilution factor 2, and the highest concentrations ranged from 0.23 to 2000 µM, depending on their solubility in DMSO. Stock solutions of mixtures were prepared using 1–3% of DMSO in DMEM, and a 3% solvent control confirmed no cytotoxicity for 72 h in each assay. All experiments were performed in triplicate using difference cell passages, limited to passages 2 to 7. During the experiments, doxorubicin (CAS No. 23214-92-8) was used as a reference chemical, and its EC50 range was consistently estimated to be between 0.2 and 0.8 µM. Cell viability was determined by dispensing 10 µL of Cell Counting Kit-8 (CCK-8 (tetrazolium salt), Dojindo, Tokyo, Japan) and incubating it for 2 h. Absorbance was then measured at a wavelength of 450 nm with a microplate reader (Infinite^®^, Tecan Co., Männedorf, Switzerland).

### 2.3. Mixtures Experimental Design

The equitoxic mixture design was used for a complex mixture toxicity test [26,27]. Once the EC10 value was derived as below 1000 µM for the single substance test, a combination of 7–10 substances was made to achieve the final EC10 level of each substance. This ensures that the cells can be exposed to EC70–EC100. For example, in the case of complex mixtures with 8 components, the highest concentration of the treatment groups was determined by calculating the sum of EC10 of A, EC10 of B, and up to EC10 of H. Subsequently, eight concentration groups were prepared with a dilution factor of 2. The conditions of the mixture toxicity assays were the same as those of the toxicity assays of single substances.

### 2.4. Data Analysis

The cell viability and cytotoxicity were calculated by Equations (1) and (2).

(1) $$\mathrm{Cell}\;\mathrm{viability}\;\left(\%\right)=\frac{\mathrm{Abs}\left(\mathrm{treatment}\right)-\mathrm{Abs}\left(\mathrm{blank}\right)}{\mathrm{Abs}\left(\mathrm{Control}\right)-\mathrm{Abs}\left(\mathrm{blank}\right)}\times 100\;\left(\%\right)$$

(2) $$\mathrm{Cytotoxicity}\;\left(\%\right)=100-\mathrm{Cell}\;\mathrm{viability}\;\left(\%\right)$$

Cell viability data were calculated by comparing with 1–3% solvent control assays. Cell viability data were analyzed by non-linear regression analysis using the SigmaPlot^®^ programme (14.5, Systat Software Inc., Palo Alto, CA, USA) to derive dose–response curves (DRCs). A sigmoid function of three parameters was fitted to all assay datasets, including three replicates of each substance. The model with the least sum of squares of residuals was selected among the sigmoid, logistic, Gompertz, Weibull, Hill, and Chapman models. The dose–response curve was then fitted to the selected model, and the EC10 value was estimated by back-calculating the equations using the ‘Solve’ option in SigmaPlot (V14.5).

The EC10 value obtained from the complex mixture test was compared with the predicted EC10 value by the CA Model, using STAGE 01 of the OpenMRA platform (https://www.openmra.org, accessed on 1 February 2024). After predicting the EC10 of the mixtures, we calculated the model deviation ratio (MDR) using Equation (3).

(3) $$\mathrm{MDR}=\frac{{\mathrm{Predicted}\;\mathrm{EC}}_{X}\;\mathrm{of}\;\mathrm{mixture}}{{\mathrm{Observed}\;\mathrm{EC}}_{X}\;\mathrm{of}\;\mathrm{mixture}}\;$$

If the predicted EC_x_ is lower than the observed EC_x_, MDR is derived as greater than 1. This indicates that the observed toxicity is stronger than the additive assumption and can be categorized as showing a synergistic effect. Similar to this way, we used MDR to determine whether the mixture toxicity has a synergistic, additive, or antagonistic effect (MDR ≤ 0.5: Antagonism, 0.5 < MDR < 2: Additivity, MDR ≥ 2: Synergism) [26].

The data analysis for cell viability and DRC fitting was applied equally to both the single and mixture toxicity tests. Additionally, the DRC model information for all mixtures are provided in the Supplementary Materials (Table S2).

## 3. Results and Discussions

To investigate the combined toxicity of different substances with varying MoAs, cytotoxicity assays were performed on fifty complex mixtures. The 25 selected chemicals were initially tested individually, followed by randomized organization of seven–ten combinations to determine any interaction effects.

### 3.1. Cytotoxicity of Single Substances

The observed EC50 and EC10 values for the individual substances are in Table 2, and the DRCs are provided in Figure S1. When comparing the EC10 values (µM), the substance with the lowest toxicity was lead chloride (EC10: 380.0 µM; EC50: 917.1 µM), followed by 2,4-DCP (EC10: 328.6 µM; EC50: 682.2 µM). On the other hand, the most toxic substance was cadmium chloride (EC10: 0.841 µM; EC50: 5.560 µM), followed by sodium selenite (EC10: 4.213 µM; EC50: 84.59 µM). Based on the single substances cytotoxicity results, we partially excluded the two most toxic substances from the further generation of complex mixture combinations because they had too limited proportions, which can lead to errors in complex mixture solution manufacturing. Se, Sb, Co, and Ni were additionally excluded from the mixture combinations due to the requirement for over 30 min of ultrasonic dispersion and poor dispersion of the mixed solution, which also can lead to experimental errors.

It is difficult to relate our observations directly to human health effects because our experimental results are calculated by measuring cell viability based on mitochondrial activity; however, the in vitro exposure concentrations of chemicals can be compared with the serum concentrations in human biomonitoring to assess their potential health effects [28,29]. In the Korean National Environmental Health Survey (KoNEHS) Cycle 3, lead and mercury were measured in whole blood of adolescents and adults. Blood lead levels were 0.802 and 1.6 µg/dL for adolescents and adults, respectively. Blood mercury levels were 1.37 and 2.75 µg/L for adolescents and adults, respectively [30]. On the other hand, the EC10 of lead (II) chloride was 105.7 mg/L, i.e., 7875 µg/dL as lead, and the EC10 of methylmercury (II) chloride was 2.9 mg/L, i.e., 2316 µg/L as mercury, which were much higher than the biomonitoring levels. The OPFRs could be compared with data from other literature, and our EC10 values of OPFRs are much higher levels, i.e., the lowest EC10 of 53.3 µM for TPhP, than the estimated plasma concentrations of OPFRs, i.e., all below 1 µM, in adult populations worldwide [31].

Although data on mixture toxicity are scarce, HepG2 cytotoxicity assay results have been reported for many single substances. For bPB, the 24 h IC50 was 643.7 µM [32], which is comparable to the EC50 of 346.2 µM, considering that we performed the chemical exposure for 48 h. For cadmium and lead, 24 h IC50s were reported as 159 and 217 µM, respectively [33], and our observations at 48 h were 5.56 and 917.1 µM. Another publication reported that 24 h EC50 for cadmium and mercury were 0.43 and 26.23 mg/L, respectively [34], and those at 48 h were 1.02 and 6.95 mg/L in our experiments. In the case of PFASs, the 24 h IC50s of PFOA, PFNA, and PFHxS were 102.0, 85.4, and 183.2 µM [35], which were consistent with the trends in relative toxicity in our study. Similarly, the 24 h TC50s were 374.7, 113.2, and 577.1 µM for PFOA, PFNA, and PFHxS, respectively [36]. There was a slight variation compared to our observation, but this may be due to different exposure periods and measurement methods; hence, the other studies used various methods, such as MTT, MTS, WST-1, or sulforhodamine B staining methods, to measure the cell viability.

To date, toxicity data for prediction model development or validation of mixture toxicity predictive models have been mainly limited to cytotoxicity test results [35,36,37,38]. This could be due to our limited knowledge in predicting toxicity, especially for chronic health effects and complex biological activity. However, we could generally find that most of our target substances exhibited disruption of energy metabolism, oxidative stress [33,39], mitochondrial membrane depolarization, necrosis, or apoptosis [32,34] in HepG2 cell lines. This indicates that the cytotoxicity of our target compounds and their mixtures may be related to further biological responses.

### 3.2. Mixture Toxicity and Their Interaction

Information on mixture composition (%) is the most important part for the development of a mixture toxicity prediction model, so we presented the randomly selected mixtures listed in Table 3. The results of mixture toxicity tests are highly dependent on the chemicals and the composition of each component. In particular, complex mixtures consist of many different chemicals, which increases the number of combinations that follow the CA model. Even if a synergistic substance is present, it is difficult to observe a synergistic effect if the synergist is present at very low levels in the mixture. For this reason, it has been suggested in the previous literature that it is appropriate to assume a CA for multicomponent mixtures [26]. All mixtures cover PFAS, heavy metals, and environmental phenols evenly. Out of fifty mixtures, five mixtures have seven components, thirty-five mixtures have eight components, nine mixtures have nine components and one mixture has ten components. Detailed mixture components and their compositions (%) are presented in Table S1.

**Figure 1:**
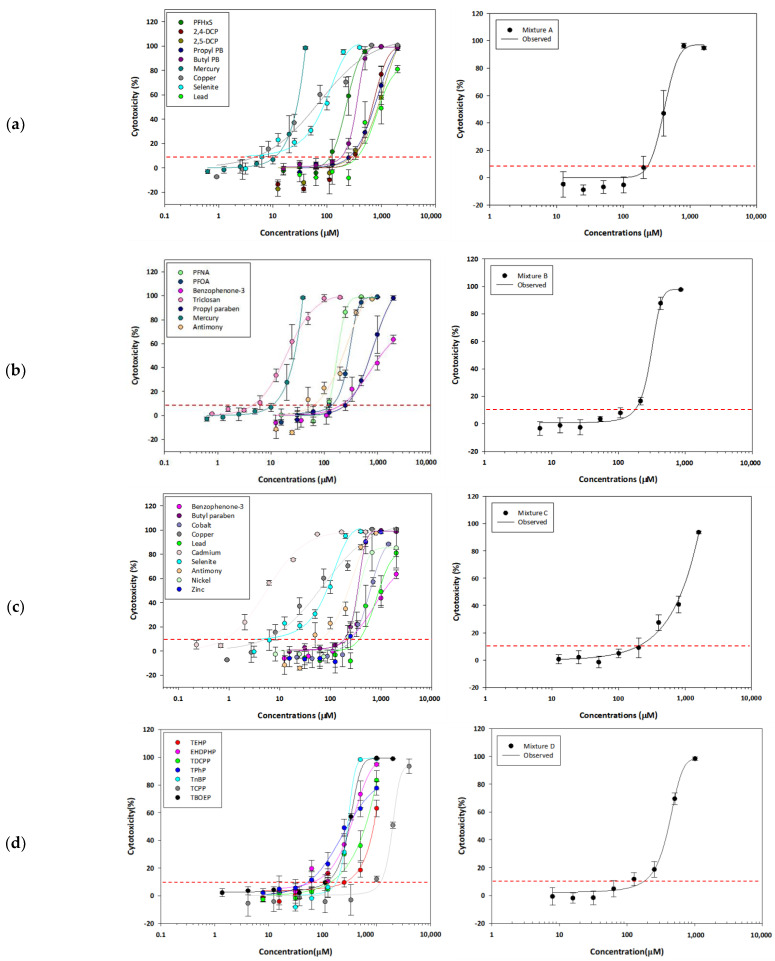
The dose–response curves of single components (**left**) and EC10-level equitoxic mixtures (**right**) for 4 representative complex mixtures: (**a**) Mixture 1; (**b**) Mixture 2; (**c**) Mixture 3; (**d**) Mixture 4. The red dash lines indicate 10% effect of cytotoxicity.

The DRCs were derived and fitted in all target complex mixtures (Table S2). Representatively, single and complex mixture DRCs were presented for four mixtures referring to the HBM4EU report (Figure 1). These four mixtures followed additivity and showed 0.86, 0.61, 0.83, and 0.82 of MDRs for Mixture 1, 2, 3, and 4, respectively (Table 3). The lowest EC10 was observed in Mixture 6, consisting of three PFASs and five OPFRs (EC10: 112.47 µM). The less toxic mixture was Mixture 47, consisting of PFHxS, 2,5-DCP, three OPFRs, and three heavy metals (EC 10: 509.95 µM).

According to the criteria for interaction effect, i.e., synergism, additivity, and antagonism, 88% of the complex mixtures showed no interaction effect. The range of MDRs was 0.31 to 1.34 (Table 3). A recent study has reported that various combinations of PFASs cause synergism in cytotoxicity assays, but only four or fewer combinations have been tested [35]. Considering this, in the case of the complex mixtures, the individual substances are essentially present at very low levels, so their toxic effects are bound to be lower. In a study validating the CA and IA models using in vitro toxicity data, it was reported that there was no interaction when the mixtures contained components with toxic effects lower than 30% [37]. In our study, the complex mixtures were designed with only 10% toxicity of each component; therefore, the additive effect following the CA model is a reasonable result.

Our experimental data showed that the CA model is more appropriate to predict the mixture toxicity values than the IA model. In a recent study, Escher et al. [37] reported that the predictions of the full IA model were indistinguishable for predicting the cytotoxicity of 200 mixtures. Overall, evidence in the literature supports the application of concentration addition as a first, protective approach. It is, therefore, also the default approach to start from in several international recommendations and frameworks [40]. The previous studies have consistently demonstrated that the CA model can be considered the dominant model for toxicity prediction of most of chemicals and their mixtures. Even if there are some substances that exhibit IA-appropriate behavior or synergistic effects, they are likely to be diluted and ultimately follow CA, especially in complex mixtures [37]. The previous literature also reported some dilemmatic situations, i.e., the observed mixture responses can be interpreted as antagonism in relation to CA and as synergisms in relation to IA, simultaneously. If the combined approach of CA and IA models should be used to cover this limitation, the stepwise modelling approach is still in a developing stage [38]. Our study aims to provide the mixture toxicity data for developing a CA/IA joint model, which will be used for the prediction of mixtures having no interaction; therefore, MDRs in our study were presented based on the CA-predicted values to maintain consistency and our study aim.

Only six mixtures have antagonistic effects in Mixtures 15, 20, 35, 36, 42, and 47. These mixtures have no significant pattern of composition or chemical differences. Considering that the average MDR of fifty mixtures was 0.68, complex mixtures tend to have an antagonistic effect due to their complex composition. In our study, only the simple biological endpoint of cell viability is measured, so it would be reasonable that the overall ability to generate cell cytotoxicity could be partially reduced by the simultaneous presence of many substances in the same mixture solution. Similarly, Caesar and Cech [41] reported that the mechanism of the antagonistic effect is unknown, but it was assumed that the target components with minor structural differences compete for the same target, leading to a reduction in overall efficacy.

Combination effects, including synergy and antagonism, may occur over a wide range of concentrations. Therefore, it is necessary to test different and various ratios of the samples [41]. In order to address this, we used an equitoxic mixture design. The equitoxic mixture design has the advantage of allowing a wide range of compositions to be tested with less effort compared to the equimolar mixture design [27]. However, experimental datasets for complex mixtures are still limited, so further validation studies and experimental datasets to compare observed and predicted toxicity values are needed. Our study has provided an experimental dataset targeting environmental phenols, heavy metal compounds, PFAS, and OPFRs. These have been chosen as representative biomonitoring examples in the HBM4EU project; however, there are still more chemicals that are exposed in the environment and consumer products [42,43,44]. Continuous efforts are required to generate and update reliable experimental data to account for exposure to unintentional mixtures.

Reliable and large mixture toxicity assay results are particularly useful in developing computational prediction models for environment or consumer products management from unintentional mixtures. Computational approaches are proven alternatives for assessing the mixtures’ toxicity due to their efficiency and convenience. The quantitative structure–activity relationship (QSAR) is the most prominent computational approach, and filling data gaps is an important part of the application of this approach [45]. In line with this research trend, our research team plans to improve and advance the conventional CA model and CA/IA joint model development in future studies. The detailed assay results, composition of mixtures, and DRC information for all single substances are provided to ensure reliable mixture prediction. For instance, we can find the MixTox package in R programme (v.1.3.2) case offers a general framework for curve fitting, mixture experimental design, and mixture toxicity prediction [46]. In the algorithm of the MixTox package, it is essential to include the exact parameters from the non-linear regression model fitting, as well as the mole fraction of each component. Providing such refined and curated data will further facilitate and support the development of computational mixture toxicity prediction models.

## 4. Conclusions

In this study, we randomly organized fifty different complex mixtures of major exogenous chemicals based on human biomonitoring data. The mixtures consisted of six environmental phenols, nine heavy metal compounds, three PFASs, and seven OPFRs. The objective was to provide a reliable and large toxicity experimental dataset of complex mixtures using HepG2 cytotoxicity assays under the same experimental conditions. Most of the mixtures exhibited additivity, consistent with previous findings on the mixture toxicity of complex mixtures with low-toxic substances. Although antagonistic effects were observed in some mixtures, we could not identify significant contributors in our experiments. Our final experimental data were formatted and added to the database (https://safety.chemdx.org/mra_db, accessed on 1 February 2024). The database is publicly available worldwide and is, therefore, expected to be used for the development of a mixture prediction model. For future work, not only the development of a mixture toxicity prediction model but also the low-level chronic effects of mixtures reflecting real-life exposures can be suggested.

## Figures and Tables

**Table 1:** Target chemicals information and four representative mixtures.

No.	Group	Chemical Name	Abbr.	CAS No.	MW
1	Phenols	2,4-dichlorophenol	2,4-DCP	120-83-2	163.0
2	Phenols	2,5-dichlorophenol	2,5-DCP	583-78-8	163.0
3	Phenols	Propyl paraben	pPB	94-13-3	180.2
4	Phenols	Butyl paraben	bPB	94-26-8	194.2
5	Phenols	Benzophenone-3	BP-3	131-57-7	228.1
6	Phenols	Triclosan	TCS	3380-34-5	289.5
7	Heavy metal	Lead chloride	Pb	7758-95-4	278.1
8	Heavy metal	Cupric sulfate	Cu	7758-98-7	249.7
9	Heavy metal	Sodium selenite	Se	10102-18-8	172.9
10	Heavy metal	Cadmium chloride hydrate	Cd	654054-66-7	183.3
11	Heavy metal	Antimony(III) chloride	Sb	10025-91-9	228.1
12	Heavy metal	Cobalt chloride	Co	7646-79-9	129.8
13	Heavy metal	Nickel dichloride	Ni	7718-54-9	129.6
14	Heavy metal	Zinc sulfate heptahydrate	Zn	7446-20-0	287.6
15	Heavy metal	Methylmercury chloride	Hg	115-09-3	251.1
16	PFASs	Perfluorooctanoic acid	PFOA	335-67-1	414.1
17	PFASs	Perfluorononanoic acid	PFNA	375-95-1	464.1
18	PFASs	Perfluorohexanesulfonic acid	PFHxS	355-46-4	438.2
19	OPFRs	Tri-n-butyl phosphate	TnBP	126-73-8	326.3
20	OPFRs	Triphenyl phosphate	TPhP	115-86-6	362.4
21	OPFRs	Tris(1-chloro-2-propyl)phosphate	TCPP	13674-84-5	398.5
22	OPFRs	2-Ethylhexyl diphenyl phosphate	EHDPHP	1241-94-7	362.4
23	OPFRs	Tris(2-butoxyethyl) phosphate	TBOEP	78-51-3	398.5
24	OPFRs	Tris (1,3-dichloropropyl) phosphate	TDCIPP	13674-87-8	430.9
25	OPFRs	Tri (2-ethylhexyl)phosphate	TEHP	78-42-2	434.6
Representative Mixtures *					
1	2,4-DCP, 2,5-DCP, pPB, bPB, Hg, Cu, Se, Pb, PFHxS				
2	pPB, BP-3, TCS, Hg, Cd, Sb, PFNA, PFOA				
3	bBP, BP-3, Co, Cu, Pb, Cd, Se, Zn, Ni				
4	TnBP, TPhP, TCPP, EHDPHP, TBOEP, TDCIPP, TEHP				

* Mixtures 1–3 refer to the ‘Mixture 2–4’ in the HBM4EU report.

**Table 2:** Observed ECx values of selected 25 single substances and dose–response curve model information in HepG2 cytotoxicity assays.

No.	Abbr.	CAS No.	EC50		EC10		Dose–Response Curve Model ^1^			
			(μM)	(mg/L)	(μM)	(mg/L)	Model	α	β	γ
1	2,4-DCP	120-83-2	682.2	111.2	328.6	53.57	Chapman	100.0	0.003	4.83
2	2,5-DCP	583-78-8	867.8	141.5	305.3	49.77	Chapman	114.7	0.001	2.26
3	pPB	94-13-3	742.4	133.8	269.8	48.62	Logistic	114.4	−2.067	839.1
4	bPB	94-26-8	346.2	67.23	191.7	37.24	Sigmoid	99.22	70.05	345.0
5	BP-3	131-57-7	1166	266.2	227.1	51.84	Logistic	76.13	−1.551	767.9
6	TCS	3380-34-5	19.29	5.590	5.041	1.460	Logistic	102.0	−1.624	19.77
7	Pb	7758-95-4	917.1	255.1	380.0	105.7	Chapman	83.33	0.002	3.901
8	Cu	7758-98-7	60.45	15.09	5.349	1.336	Logistic	107.4	−0.882	70.68
9	Se	10102-18-8	84.59	14.63	4.213	0.729	Gompertz	102.2	68.15	61.69
10	Cd	654054-66-7	5.560	1.020	0.841	0.154	Logistic	99.76	−1.165	5.537
11	Sb	10025-91-9	225.7	51.49	69.33	15.82	Chapman	104.9	0.005	1.932
12	Co	7646-79-9	612.0	79.46	272.7	35.41	Chapman	91.63	0.003	4.234
13	Ni	7718-54-9	372.4	48.27	213.6	27.68	Chapman	85.17	0.008	11.59
14	Zn	7446-20-0	336.9	96.88	243.0	69.88	Chapman	98.29	0.013	50.16
15	Hg	115-09-3	27.68	6.950	11.55	2.900	Logistic	27675	−1.843	851.1
16	PFOA	335-67-1	290.4	120.2	150.4	62.29	Sigmoid	98.52	63.31	288.5
17	PFNA	375-95-1	176.8	82.04	122.6	56.88	Chapman	98.92	0.022	32.37
18	PFHxS	355-46-4	223.4	97.87	117.6	51.54	Chapman	99.10	0.010	6.501
19	TnBP ^2^	126-73-8	270.5	72.05	167.4	44.58	Sigmoid	98.90	46.72	269.5
20	TPhP	115-86-6	288.0	93.98	53.32	17.40	Logistic	0.961	87.47	−1.385
21	TCPP	13674-84-5	306.6	100.4	175.8	57.59	Chapman	0.937	100.9	0.001
22	EHDPHP	1241-94-7	327.0	118.5	86.71	31.42	Gompertz	0.965	96.56	194.2
23	TBOEP	78-51-3	308.6	123.0	119.9	47.76	Sigmoid	0.995	99.19	85.59
24	TDCIPP	13674-87-8	617.8	266.2	142.2	61.28	Logistic	0.940	971.3	−1.125
25	TEHP	78-42-2	878.4	381.8	265.8	115.5	Gompertz	0.858	493.5	1150

^1^ All the dataset was applied nonlinear regression method with 3-parameter sigmoidal models (sigmoid, logistic, Chapman, Weibull, Hill and Gompertz). The final model equations in Table 2 are below: sigmoid: f = a/(1 + exp(−(x − x0)/b)); logistic: f = if(x <= 0, if(b < 0, 0, a), if(b > 0, a/(1 + abs(x/x0)^b), a × abs((x/x0))^(abs(b))/(1 + (abs(x/x0))^(abs(b))))); Chapman: f = a × (1 − exp(−b × x))^c; Gompertz: f = a × exp(−exp(−(x − x0)/b)). ^2^ Toxicity data for OPFRs are cited from Kim et al. [21].

**Table 3:** The observed EC10 and model deviation ratio (MDR) of complex mixtures consisting of selected chemicals from the biomonitoring data analysis report in HepG2 cytotoxicity assays.

Mixtures	EC10 (μM)	MDR	Interaction	Mixtures	EC10 (μM)	MDR	Interaction
1	208.0	0.86	Additivity	26	348.0	0.53	Additivity
2	175.8	0.61	Additivity	27	296.0	0.96	Additivity
3	194.6	0.83	Additivity	28	338.7	0.54	Additivity
4	175.3	0.82	Additivity	29	267.8	0.58	Additivity
5	175.3	1.09	Additivity	30	160.9	0.50	Additivity
6	112.5	0.94	Additivity	31	164.6	0.61	Additivity
7	161.3	0.83	Additivity	32	216.6	0.58	Additivity
8	244.5	0.56	Additivity	33	272.1	0.52	Additivity
9	331.8	0.56	Additivity	34	157.6	0.53	Additivity
10	223.6	0.53	Additivity	35	430.2	0.45	Antagonism
11	263.4	0.67	Additivity	36	504.7	0.31	Antagonism
12	126.0	0.95	Additivity	37	114.2	1.14	Additivity
13	154.0	0.73	Additivity	38	171.6	0.60	Additivity
14	241.5	0.51	Additivity	39	160.9	0.85	Additivity
15	264.2	0.44	Antagonism	40	209.6	0.55	Additivity
16	205.5	1.18	Additivity	41	232.2	0.66	Additivity
17	240.4	0.79	Additivity	42	215.9	0.48	Antagonism
18	185.4	1.03	Additivity	43	220.1	0.59	Additivity
19	189.6	0.77	Additivity	44	241.0	0.50	Additivity
20	504.5	0.37	Antagonism	45	255.1	0.51	Additivity
21	120.4	1.34	Additivity	46	252.0	0.54	Additivity
22	223.5	0.80	Additivity	47	510.0	0.33	Antagonism
23	190.3	0.99	Additivity	48	181.2	0.63	Additivity
24	292.6	0.58	Additivity	49	264.0	0.53	Additivity
25	222.7	0.71	Additivity	50	345.8	0.57	Additivity

## Data Availability

All datasets are publicly available at Safe and Sustainable by Design Platfom in ChemDX (https://safety.chemdx.org/combi_tox, accessed on 1 February 2024) by searching the single substance information in ‘Mixture’ tap. The complex mixture test dataset can be referenced by starting with CSRC (e.g., CSRC135_1545 and CSRC132_1542).

## Supplementary Materials

The following supporting information can be downloaded at https://www.mdpi.com/article/10.3390/toxics12020126/s1, Table S1: Fifty kinds of mixture combinations in this study, Table S2: Dose–response curve model information of target complex mixtures in HepG2 cytotoxicity assays, Figure S1: Dose–response curves of target single substances.

## References

[1] ECHA Biocidal Products Regulation. https://echa.europa.eu/regulations/biocidal-products-regulation/legislation.

[2] Ministry of Environment (MOE) (2018). Act No. 15511 of the Korean Ministry of Environment of the Council of 20 March 2018 Concerning Household Chemical Products and Biocidal Products Safety.

[3] Martin O., Scholze M., Ermler S., McPhie J., Bopp S.K., Kienzler A., Parissis N., Kortenkamp A. (2021). Ten years of research on synergisms and antagonisms in chemical mixtures: A systematic review and quantitative reappraisal of mixture studies. Environ. Int..

[4] Kortenkamp A. (2014). Low dose mixture effects of endocrine disrupters and their implications for regulatory thresholds in chemical risk assessment. Curr. Opin. Pharmacol..

[5] Kortenkamp A., Backhaus T., Faust M. (2009). State of the Art Report on Mixture Toxicity—Final Report.

[6] Loewe S., Muischnek H. (1926). Über Kombinationswirkungen. Naunyn-Schmiedebergs Arch. Exp. Pathol. Pharmakol..

[7] Bliss C.I. (1939). The toxicity of poisons applied jointly. Ann. Appl. Biol..

[8] Kortenkamp A., Vinggaard A.M., Mengelers M., Slama R., Silva M.J., Louro H., Viegas S., Tavares A., Goen T., Ermler S. (2021). Deliverable 15.5 Case Study Reports on Mixture Health Effects.

[9] Valerio L.G. (2009). In silico toxicology for the pharmaceutical sciences. Toxicol. Appl. Pharmacol..

[10] Howard G.J., Webster T.F. (2009). Generalized concentration addition: A method for examining mixtures containing partial agonists. J. Theor. Biol..

[11] Altenburger R., Walter H., Grote M. (2004). What contributes to the combined effect of a complex mixture?. Environ. Sci. Technol..

[12] Ermler S., Scholze M., Kortenkamp A. (2014). Genotoxic mixtures and dissimilar action: Concepts for prediction and assessment. Arch. Toxicol..

[13] Ezechias M., Cajthaml T. (2016). Novel full logistic model for estimation of the estrogenic activity of chemical mixtures. Toxicology.

[14] Kim J., Seo M., Choi J., Na M. (2022). MRA Toolbox v. 1.0: A web-based toolbox for predicting mixture toxicity of chemical substances in chemical products. Sci. Rep..

[15] Baas J., Willems J., Jager T., Kraak M.H., Vandenbrouck T., Kooijman S.A. (2009). Prediction of daphnid survival after in situ exposure to complex mixtures. Environ. Sci. Technol..

[16] Worth A., Kienzler A., Bopp S., Joint Research Centre, Institute for Health and Consumer Protection (2015). Scientific Methodologies for the Assessment of Combined Effects—A Survey and Literature Review.

[17] Donnelly K.C., Lingenfelter R., Cizmas L., Falahatpisheh M.H., Qian Y., Tang Y., Garcia S., Ramos K., Tiffany-Castiglioni E., Mumtaz M.M. (2004). Toxicity assessment of complex mixtures remains a goal. Environ. Toxicol. Pharmacol..

[18] Sobus J.R., DeWoskin R.S., Tan Y.M., Pleil J.D., Phillips M.B., George B.J., Christensen K., Schreinemachers D.M., Williams M.A., Hubal E.A. (2015). Uses of NHANES Biomarker Data for Chemical Risk Assessment: Trends, Challenges, and Opportunities. Env. Health Perspect..

[19] Luijten M., Vlaanderen J., Kortenkamp A., Antignac J.P., Barouki R., Bil W., van den Brand A., den Braver-Sewradj S., van Klaveren J., Mengelers M. (2023). Mixture risk assessment and human biomonitoring: Lessons learnt from HBM4EU. Int. J. Hyg. Environ. Health.

[20] Vlaanderen J., Ottenbros I., Crépet A., Trocellier L., Lebret E., Bogers R., Vermeulen R., Roth C., Govarts E. (2019). Deliverable Report D15.3. Report Real-Life Exposure Profiles from Re-Analysis of Existing HBM Mixture Data.

[21] Kim S., Kang K., Kim J., Na M., Choi J. (2023). Toxicity of organophosphorus flame retardants (OPFRs) and their mixtures in *Vibrio fischeri* and human hepatocyte HepG2. J. Environ. Health Sci..

[22] An J., Hu J., Shang Y., Zhong Y., Zhang X., Yu Z. (2016). The cytotoxicity of organophosphate flame retardants on HepG2, A549 and Caco-2 cells. J. Environ. Sci. Health A Toxic Hazard. Subst. Environ. Eng..

[23] Jo A., Kim S., Ji K., Kho Y., Choi K. (2020). Influence of Vegetarian Dietary Intervention on Urinary Paraben Concentrations: A Pilot Study with ‘Temple Stay’ Participants. Toxics.

[24] Rasmussen P.E., Levesque C., Chenier M., Gardner H.D., Jones-Otazo H., Petrovic S. (2013). Canadian House Dust Study: Population-based concentrations, loads and loading rates of arsenic, cadmium, chromium, copper, nickel, lead, and zinc inside urban homes. Sci. Total Environ..

[25] Plichta V., Steinwider J., Vogel N., Weber T., Kolossa-Gehring M., Murinova L.P., Wimmerova S., Tratnik J.S., Horvat M., Koppen G. (2022). Risk Assessment of Dietary Exposure to Organophosphorus Flame Retardants in Children by Using HBM-Data. Toxics.

[26] Belden J.B., Gilliom R.J., Lydy M.J. (2007). How well can we predict the toxicity of pesticide mixtures to aquatic life?. Integr. Environ. Assess. Manag..

[27] Faust M., Altenburger R., Backhaus T., Blanck H., Boedeker W., Gramatica P., Hamer V., Scholze M., Vighi M., Grimme L.H. (2001). Predicting the joint algal toxicity of multi-component s-triazine mixtures at low-effect concentrations of individual toxicants. Aquat. Toxicol..

[28] Li C.H., Ren X.M., Guo L.H. (2019). Adipogenic activity of oligomeric hexafluoropropylene oxide (Perfluorooctanoic acid alternative) through peroxisome proliferator-activated receptor γ pathway. Environ. Sci. Technol..

[29] Li C.H., Ren X.M., Ruan T., Cao L.Y., Xin Y., Guo L.H., Jiang G. (2018). Chlorinated polyfluorinated ether sulfonates exhibit higher activity toward peroxisome proliferator-activated receptors signaling pathways than perfluorooctanesulfonate. Environ. Sci. Technol..

[30] Jung S.K., Choi W., Kim S.Y., Hong S., Jeon H.L., Joo Y., Lee C., Choi K., Kim S., Lee K.J. (2022). Profile of environmental chemicals in the Korean population—Results of the Korean National Environmental Health Survey (KoNEHS) Cycle 3, 2015–2017. Int. Environ. Res. Public Health.

[31] Blum A., Behl M., Birnbaum L., Diamond M.L., Phillips A., Singla V., Sipes N.S., Stapleton H.M., Venier M. (2019). Organophosphate ester flame retardants: Are they a regrettable substitution for polybrominated diphenyl ethers?. Environ. Sci Technol. Lett..

[32] Kizhedath A., Wilkinson S., Glassey J. (2019). Assessment of hepatotoxicity and dermal toxicity of butyl paraben and methyl paraben using HepG2 and HDFn in vitro models. Toxicol. Vitro.

[33] Muthusamy S., Peng C., Ng J.C. (2016). Effects of binary mixtures of benzo[a]pyrene, arsenic, cadmium, and lead on oxidative stress and toxicity in HepG2 cells. Chemosphere.

[34] Cordier W., Yousaf M., Nell M.J., Steenkamp V. (2021). Underlying mechanisms of cytotoxicity in HepG2 hepatocarcinoma cells exposed to arsenic, cadmium and mercury individually and in combination. Toxicol. Vitro.

[35] Ojo A.F., Peng C., Ng J.C. (2020). Combined effects and toxicological interactions of perfluoroalkyl and polyfluoroalkyl substances mixtures in human liver cells (HepG2). Environ. Pollut..

[36] Amstutz V.H., Cengo A., Gehres F., Sijm D., Vrolijk M.F. (2022). Investigating the cytotoxicity of per- and polyfluoroalkyl substances in HepG2 cells: A structure-activity relationship approach. Toxicology.

[37] Escher B., Braun G., Zarfl C. (2020). Exploring the concepts of concentration addition and independent action using a linear low-effect mixture model. Environ. Toxicol. Chem..

[38] Altenburger R., Scholze M., Busch W., Escher B.I., Jakobs G., Krauss M., Kruger J., Neale P.A., Ait-Aissa S., Almeida A.C. (2018). Mixture effects in samples of multiple contaminants—An inter-laboratory study with manifold bioassays. Environ. Int..

[39] Szelag S., Zablocka A., Trzeciak K., Drozd A., Baranowska-Bosiacka I., Kolasa A., Goschorska M., Chlubek D., Gutowska I. (2016). Propylparaben-induced disruption of energy metabolism in human HepG2 cell line leads to increased synthesis of superoxide anions and apoptosis. Toxicol. Vitro.

[40] Bopp S.K., Barouki R., Brack W., Dalla Costa S., Dorne J.C.M., Drakvik P.E., Faust M., Karjalainen T.K., Kephalopoulos S., van Klaveren J. (2018). Current EU research activities on combined exposure to multiple chemicals. Environ. Int..

[41] Caesar L.K., Cech N.B. (2019). Synergy and antagonism in natural product extracts: When 1 + 1 does not equal 2. Nat. Prod. Rep..

[42] Lee I., Ji K. (2022). Identification of combinations of endocrine disrupting chemicals in household chemical products that require mixture toxicity testing. Ecotoxicol. Environ. Saf..

[43] Gabb H.A., Blake C. (2016). An Informatics Approach to Evaluating Combined Chemical Exposures from Consumer Products: A Case Study of Asthma-Associated Chemicals and Potential Endocrine Disruptors. Environ. Health Perspect..

[44] Kim S., Seo M., Na M., Kim J. (2021). Investigation on Combined Inhalation Exposure Scenarios to Biocidal Mixtures: Biocidal and Household Chemical Products in South Korea. Toxics.

[45] Kar S., Leszczynski J. (2022). Computational approaches in assessments of mixture toxicity. Curr. Opin. Toxicol..

[46] Zhu X.W., Chen J.Y. (2016). Mixtox: An R package for mixture toxicity assessment. R J..

