# Supplementary material for: In Vitro Toxicity Screening of Fifty Complex Mixtures in HepG2 Cells
Source: Toxics. 2024 Feb 2;12(2):126. doi: 10.3390/toxics12020126 (PMC10892977; doi:10.3390/toxics12020126)
Supplement: Supplementary file 1 [file toxics-12-00126-s001.zip › toxics-2823598-supplementary.pdf]

Supplementary Materials

In vitro toxicity screening of fifty complex mixtures in HepG2 cells

Sunmi Kim [1,*], Kyounghee Kang [1], Haena Kim [1,2] and Myungwon Seo [1]

Table S1. Fifty kinds of mixture combinations in this study.

**Mixtures**		**Chemicals and their compositions (%) in complex mixture**									
Mixture 1	Chemicals	PFHxS	2,4DCP	2,5DCP	pPB	bPB	Hg	Cu	Se	Pb	
(n = 9)	MF* (%)	7.286	20.358	18.915	16.714	11.878	0.716	0.331	0.261	23.540	
Mixture 2	Chemicals	PFNA	PFOA	BP-3	TCS	pPB	Hg	Cd	Sb		
(n = 8)	MF (%)	14.304	17.557	26.524	0.588	31.488	1.348	0.098	8.092		
Mixture 3	Chemicals	BP-3	bPB	Co	Cu	Pb	Cd	Se	Zn	Sb	Ni
(n = 10)	MF (%)	14.133	11.924	16.960	0.333	23.631	0.052	0.262	15.112	4.312	13.282
Mixture 4	Chemicals	TEHP	TDCPP	EHDPHP	TPhP	TnBP	TCPP	TBOEP			
(n = 7)	MF (%)	26.287	14.065	8.575	5.273	16.557	17.389	11.854			
Mixture 5	Chemicals	2,4-DCP	2,5-DCP	pPB	bPB	BP-3	TCS	TnBP	TPhP	TCPP	
(n = 9)	MF (%)	19.058	17.707	15.646	11.120	13.179	0.292	9.708	3.093	10.196	
Mixture 6	Chemicals	PFNA	PFOA	PFHxS	EHDPHP	TBOEP	TDCPP	TEHP	TPhP		
(n = 8)	MF (%)	14.451	17.738	13.868	10.224	14.134	16.769	6.529	6.287		
Mixture 7	Chemicals	Co	Cu	Pb	Cd	Se	Zn	Sb	Hg	Ni	
(n = 6)	MF (%)	22.715	0.446	31.651	0.070	0.351	20.240	5.774	0.962	17.790	
Mixture 8	Chemicals	Pb	Cd	Zn	Hg	TBOEP	TDCPP	TEHP			
(n = 7)	MF (%)	39.880	0.088	25.503	1.212	12.580	14.926	5.811			
Mixture 9	Chemicals	PFNA	PFOA	PFHxS	2,4-DCP	2,5-DCP	pPB	bPB	BP-3	TCS	
(n = 9)	MF (%)	7.133	8.755	6.845	19.125	17.770	15.701	11.159	13.218	0.293	
Mixture 10	Chemicals	PFNA	PFOA	PFHxS	Co	Cu	Se	Sb	Ni		
(n = 8)	MF (%)	12.823	15.739	12.305	28.533	0.560	0.441	7.253	22.346		
Mixture 11	Chemicals	PFOA	PFHxS	Pb	Hg	Zn	TDCPP	TPhP	2,5-DCP	bPB	
(n = 9)	MF (%)	9.430	7.373	23.821	0.724	15.233	8.916	3.343	19.140	12.019	
Mixture 12	Chemicals	PFNA	PFHxS	Co	Se	Hg	TnBP	TDCPP			
(n = 7)	MF (%)	14.620	14.030	32.533	0.503	1.378	19.971	16.966			
Mixture 13	Chemicals	PFHxS	BP-3	TCS	TnBP	EHDPHP	TBOEP	Cd	Sb	Ni	
(n = 9)	MF (%)	11.673	22.543	0.500	16.616	8.606	11.898	0.083	6.881	21.199	
Mixture 14	Chemicals	PFOA	2,5-DCP	TCS	TBOEP	TDCPP	Zn	Cu	Hg		
(n = 8)	MF (%)	15.307	31.067	0.513	12.197	14.471	24.726	0.544	1.175		
Mixture 15	Chemicals	2,4-DCP	Cd	Se	Zn	Sb	EHDPHP	TBOEP	TDCPP	TEHP	
(n = 9)	MF (%)	31.292	0.080	0.401	23.139	6.601	8.257	11.414	13.543	5.272	
Mixture 16	Chemicals	2,4-DCP	2,5-DCP	BP-3	Cd	Ni	Pb	Zn			
(n = 7)	MF (%)	19.348	17.976	13.372	0.050	12.575	22.373	14.307			
Mixture 17	Chemicals	PFHxS	BP-3	pPB	TnBP	TPhP	Pb	Co	Cu	Ni	
(n = 9)	MF (%)	6.890	13.306	15.806	9.808	3.124	22.263	15.977	0.313	12.513	

Mixture 18	Chemicals	PFNA	PFOA	PFHxS	2,5-DCP	BP-3	Se	Ni	Pb	
(n = 8)	MF (%)	8.059	9.891	7.733	20.076	14.934	0.277	14.043	24.986	
Mixture 19	Chemicals	bPB	2,5-DCP	TCS	TBOEP	TCPP	TEHP	Zn	Ni	Hg
(n = 9)	MF (%)	14.511	23.108	0.382	9.072	13.307	4.191	18.391	16.164	0.874
Mixture 20	Chemicals	PFHxS	PFNA	pPB	Zn	Ni	TCPP	TnBP		
(n = 7)	MF (%)	8.980	9.357	20.598	18.553	16.307	13.424	12.782		
Mixture 21	Chemicals	PFHxS	Pb	PFNA	Co	TPhP	EHDPHP	TBOEP	TDCPP	
(n = 8)	MF (%)	9.082	29.343	9.464	21.059	4.117	6.696	9.256	10.982	
Mixture 22	Chemicals	PFHxS	2,4-DCP	2,5-DCP	bPB	BP-3	TPhP	EHDPHP	TBOEP	
(n = 8)	MF (%)	8.223	22.975	21.347	13.405	15.879	3.728	6.062	8.381	
Mixture 23	Chemicals	PFHxS	2,4-DCP	2,5-DCP	pPB	TCS	TnBP	TCPP	TDCPP	
(n = 8)	MF (%)	7.779	21.736	20.196	17.845	0.333	11.073	11.630	9.407	
Mixture 24	Chemicals	2,4-DCP	pPB	Sb	TnBP	TCPP	EHDPHP	TBOEP	TDCPP	
(n = 8)	MF (%)	24.167	19.841	5.098	12.312	12.930	6.377	8.815	10.459	
Mixture 25	Chemicals	2,5-DCP	pPB	Sb	PFOA	TCS	Zn	EHDPHP	TDCPP	
(n = 8)	MF (%)	24.007	21.212	5.451	11.828	0.396	19.106	6.817	11.182	
Mixture 26	Chemicals	PFHxS	2,4-DCP	pPB	Zn	TnBP	TPhP	TCPP	TBOEP	
(n = 8)	MF (%)	7.971	22.273	18.285	16.470	11.347	3.614	11.917	8.124	
Mixture 27	Chemicals	2,4-DCP	2,5-DCP	PFNA	Zn	TCPP	EHDPHP	TBOEP	TDCPP	
(n = 8)	MF (%)	21.561	20.033	8.041	15.944	11.536	5.689	7.865	9.331	
Mixture 28	Chemicals	2,4-DCP	2,5-DCP	Sb	PFNA	PFOA	BP-3	Ni	TEHP	
(n = 8)	MF (%)	22.320	20.738	4.709	8.324	10.217	15.426	14.506	3.761	
Mixture 29	Chemicals	2,5-DCP	PFNA	PFOA	Co	Zn	TDCPP	Cu	Cd	
(n = 8)	MF (%)	24.575	9.864	12.108	21.950	19.558	11.447	0.431	0.068	
Mixture 30	Chemicals	PFHxS	PFNA	TCS	Ni	TnBP	Hg	Cu	Cd	
(n = 8)	MF (%)	18.264	19.033	0.783	33.167	25.998	1.794	0.831	0.131	
Mixture 31	Chemicals	PFHxS	Sb	TCS	Co	Ni	TBOEP	Cu	Cd	
(n = 8)	MF (%)	14.622	8.619	0.627	33.906	26.553	14.903	0.665	0.105	
Mixture 32	Chemicals	Sb	Co	TnBP	TPhP	TCPP	TBOEP	TDCPP	Cd	
(n = 8)	MF (%)	6.922	27.230	16.716	5.324	17.556	11.969	14.200	0.084	
Mixture 33	Chemicals	PFHxS	Sb	PFNA	Zn	TnBP	TDCPP	TEHP	Hg	
(n = 8)	MF (%)	10.321	6.084	10.756	21.326	14.692	12.481	23.326	1.014	
Mixture 34	Chemicals	bPB	TCS	TnBP	TPhP	EHDPHP	TDCPP	Hg	Cu	
(n = 8)	MF (%)	28.905	0.760	25.238	8.038	13.071	21.440	1.741	0.806	
Mixture 35	Chemicals	PFHxS	2,4-DCP	2,5-DCP	bPB	TCS	Co	Ni	TBOEP	
(n = 8)	MF (%)	7.566	21.140	19.642	12.334	0.324	17.544	13.739	7.711	
Mixture 36	Chemicals	PFHxS	2,4-DCP	Sb	TCS	Zn	Ni	TCPP	EHDPHP	
(n = 8)	MF (%)	9.487	26.508	5.592	0.407	19.602	17.228	14.183	6.994	
Mixture 37	Chemicals	bPB	Sb	PFNA	BP-3	TnBP	TBOEP	TDCPP	Cd	
(n = 8)	MF (%)	18.417	6.659	11.772	21.816	16.081	11.514	13.661	0.081	
Mixture 38	Chemicals	PFHxS	2,4-DCP	bPB	TnBP	TPhP	Hg	Cu	Cd	
(n = 8)	MF (%)	8.371	39.681	23.152	20.215	6.438	1.395	0.646	0.102	
Mixture 39	Chemicals	PFNA	PFOA	TnBP	TCPP	EHDPHP	TBOEP	TEHP	Cd	

(n = 8)	MF (%)	11.250	13.808	15.367	16.139	7.959	11.003	24.398	0.077
Mixture 40	Chemicals	Sb	PFOA	BP-3	TCS	Zn	TnBP	TPhP	Hg
(n = 8)	MF (%)	7.477	16.224	24.495	0.544	26.208	18.056	5.751	1.246
Mixture 41	Chemicals	PFHxS	Sb	PFOA	Zn	Ni	TnBP	TBOEP	TDCPP
(n = 8)	MF (%)	9.613	5.666	12.296	19.862	17.457	13.684	9.798	11.624
Mixture 42	Chemicals	PFHxS	BP-3	TCS	Ni	TnBP	EHDPHP	Hg	Cu
(n = 8)	MF (%)	14.096	27.221	0.604	25.597	20.065	10.392	1.384	0.641
Mixture 43	Chemicals	2,4-DCP	Sb	PFOA	BP-3	TCS	TBOEP	TDCPP	Cd
(n = 8)	MF (%)	31.493	6.644	14.416	21.766	0.483	11.488	13.629	0.081
Mixture 44	Chemicals	PFHxS	Sb	Zn	Ni	TPhP	TBOEP	TDCPP	Cd
(n = 8)	MF (%)	12.254	7.223	25.319	22.253	5.556	12.489	14.818	0.088
Mixture 45	Chemicals	bPB	PFNA	PFOA	BP-3	EHDPHP	TEHP	Cu	Cd
(n = 8)	MF (%)	18.251	11.666	14.319	21.620	8.254	25.301	0.509	0.080
Mixture 46	Chemicals	PFHxS	Sb	Co	Zn	Ni	TCPP	EHDPHP	TDCPP
(n = 8)	MF (%)	8.903	5.248	20.645	18.396	16.168	13.310	6.564	10.766
Mixture 47	Chemicals	PFHxS	2,5-DCP	Co	Zn	TPhP	TCPP	TEHP	Hg
(n = 8)	MF (%)	8.138	21.128	18.871	16.815	3.690	12.167	18.392	0.799
Mixture 48	Chemicals	2,5-DCP	TCS	Pb	EHDPHP	TBOEP	Hg	Cu	Cd
(n = 8)	MF (%)	33.381	0.551	41.544	9.479	13.105	1.263	0.585	0.092
Mixture 49	Chemicals	2,5-DCP	bPB	PFNA	PFOA	TCS	EHDPHP	TBOEP	TDCPP
(n = 8)	MF (%)	27.167	17.060	10.905	13.385	0.449	7.715	10.666	12.654
Mixture 50	Chemicals	2,4-DCP	Sb	PFNA	PFOA	BP-3	Pb	TCPP	TBOEP
(n = 8)	MF (%)	20.881	4.405	7.788	9.559	14.432	24.146	11.172	7.617

*MF: Mole fraction (moles of single substance divided by total moles of mixture solution).

Table S2. Dose–response curve model information of target complex mixtures in HepG2 cytotoxicity assays.

No.	Dose–Response Curve model[1]				
	**Model**	**α**	**β**	**γ**	**Adjusted R^2**
Mixture 1	Gompertz	97.32	152.7	338.3	0.950
Mixture 2	Sigmoid	97.98	59.02	304.2	0.985
Mixture 3	Chapman	613.1	0.0003	2.150	0.970
Mixture 4	Sigmoid	98.50	107.4	409.5	0.976
Mixture 5	Gompertz	103.0	262.0	294.0	0.960
Mixture 6	Sigmoid	101.0	56.40	237.0	0.985
Mixture 7	Sigmoid	101.0	100.0	382.0	0.977
Mixture 8	Sigmoid	97.10	54.90	363.0	0.974
Mixture 9	Sigmoid	99.34	79.38	505.7	0.980
Mixture 10	Logistic	103.0	4.23	379.0	0.961
Mixture 11	Sigmoid	98.40	105.0	491.0	0.962
Mixture 12	Gompertz	103.0	72.10	187.0	0.971
Mixture 13	Sigmoid	102.0	66.70	302.0	0.991
Mixture 14	Sigmoid	99.60	26.30	299.0	0.978
Mixture 15	Hill	98.60	8.70	340.0	0.980

Mixture 16	Hill	6.51E+06	1.06	6.35E+07	0.941
Mixture 17	Sigmoid	104.0	148.0	572.0	0.973
Mixture 18	Sigmoid	129.0	367.0	1090	0.962
Mixture 19	Gompertz	102.0	94.80	400.0	0.981
Mixture 20	Gompertz	105.0	218.8	691.6	0.963
Mixture 21	Sigmoid	102.0	100.9	344.4	0.985
Mixture 22	Sigmoid	100.0	60.80	357.0	0.988
Mixture 23	Gompertz	101.0	118.0	289.0	0.984
Mixture 24	Sigmoid	99.20	47.60	397.0	0.986
Mixture 25	Sigmoid	98.50	51.90	336.0	0.990
Mixture 26	Hill	102.0	7.28	472.0	0.984
Mixture 27	Sigmoid	98.67	48.33	401.5	0.987
Mixture 28	Chapman	99.60	0.01	34.50	0.989
Mixture 29	Hill	100.4	8.29	349.1	0.989
Mixture 30	Chapman	101.0	0.03	533.0	0.985
Mixture 31	Chapman	101.0	0.01	14.10	0.987
Mixture 32	Sigmoid	101.0	43.20	312.0	0.984
Mixture 33	Sigmoid	97.20	29.80	337.0	0.989
Mixture 34	Sigmoid	99.30	21.90	206.0	0.987
Mixture 35	Chapman	101.3	0.01	22.40	0.972
Mixture 36	Chapman	98.57	0.01	463.7	0.951
Mixture 37	Sigmoid	99.60	55.00	235.0	0.986
Mixture 38	Sigmoid	100.0	35.00	249.0	0.988
Mixture 39	Sigmoid	101.0	71.80	318.0	0.989
Mixture 40	Sigmoid	98.90	41.50	300.0	0.989
Mixture 41	Sigmoid	98.48	58.61	359.9	0.978
Mixture 42	Chapman	100.3	0.03	1345	0.955
Mixture 43	Hill	99.20	8.52	284.5	0.984
Mixture 44	Chapman	98.59	0.02	182.2	0.967
Mixture 45	Chapman	98.87	0.04	1.82E+04	0.984
Mixture 46	Sigmoid	101.6	79.8	428.7	0.975
Mixture 47	Chapman	102.2	0.005	24.34	0.966
Mixture 48	Sigmoid	99.74	37.17	262.7	0.980
Mixture 49	Logistic	99.02	27.68	285.7	0.981
Mixture 50	Hill	97.83	7.20	467.5	0.983

[1] All the dataset was applied nonlinear regression method with 3-parameter sigmoidal models (sigmoid, logistic, Chapman, Weibull, Hill, and Gompertz). The final model equations in Table 2 are below: sigmoid: f = a/(1+exp(-(x-x0)/b)); logistic: f = if(x<=0, if(b<0,0,a), if(b>0, a/(1+abs(x/x0)^b), a*abs((x/x0))^(abs(b))/(1+(abs(x/x0))^(abs(b))))); Chapman: f = a*(1-exp(-b*x))^c; Gompertz: f = a*exp(-exp(-(x-x0)/b)).

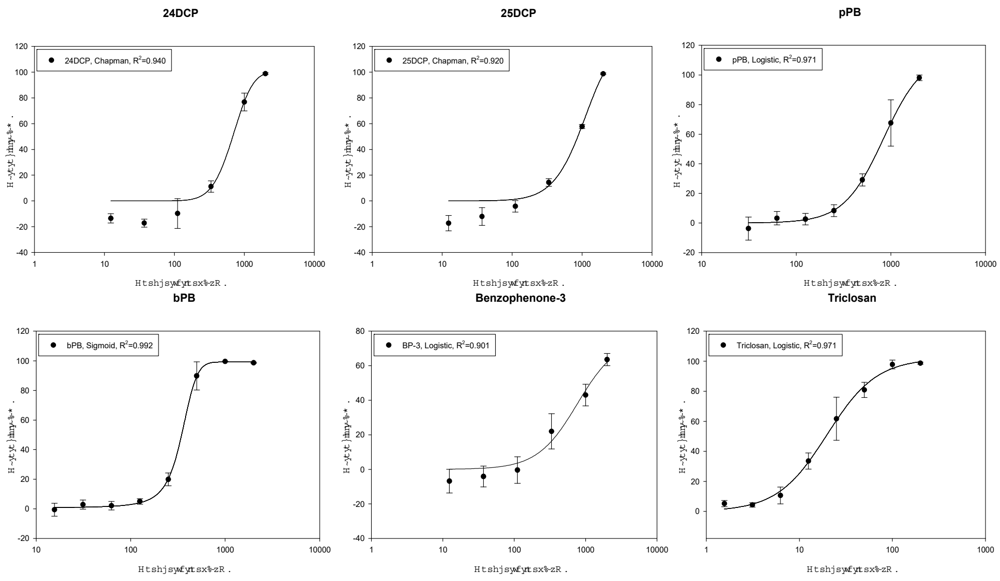
24DCP
24DCP, Chapman, R^2=0.940
25DCP
25DCP, Chapman, R^2=0.920
pPB
pPB, Logistic, R^2=0.971
bPB
bPB, Sigmoid, R^2=0.992
Benzophenone-3
BP-3, Logistic, R^2=0.901
Triclosan
Triclosan, Logistic, R^2=0.971

(a)

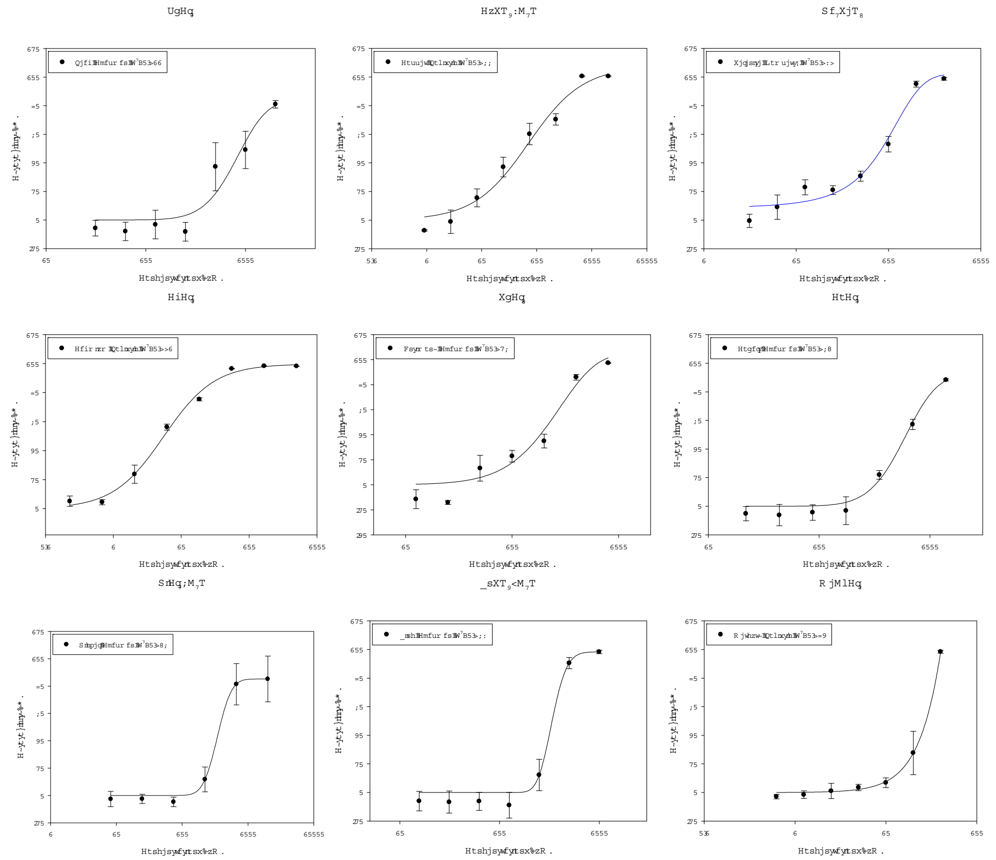

(b)

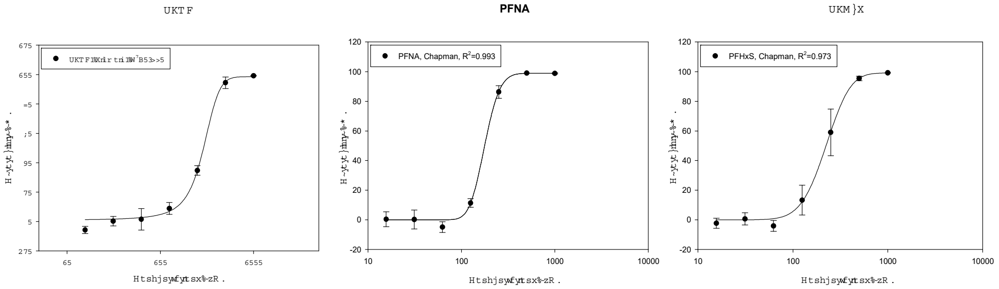
PFNA
PFNA, Chapman, R^2=0.993
PFHxS, Chapman, R^2=0.973

(c)

(d)

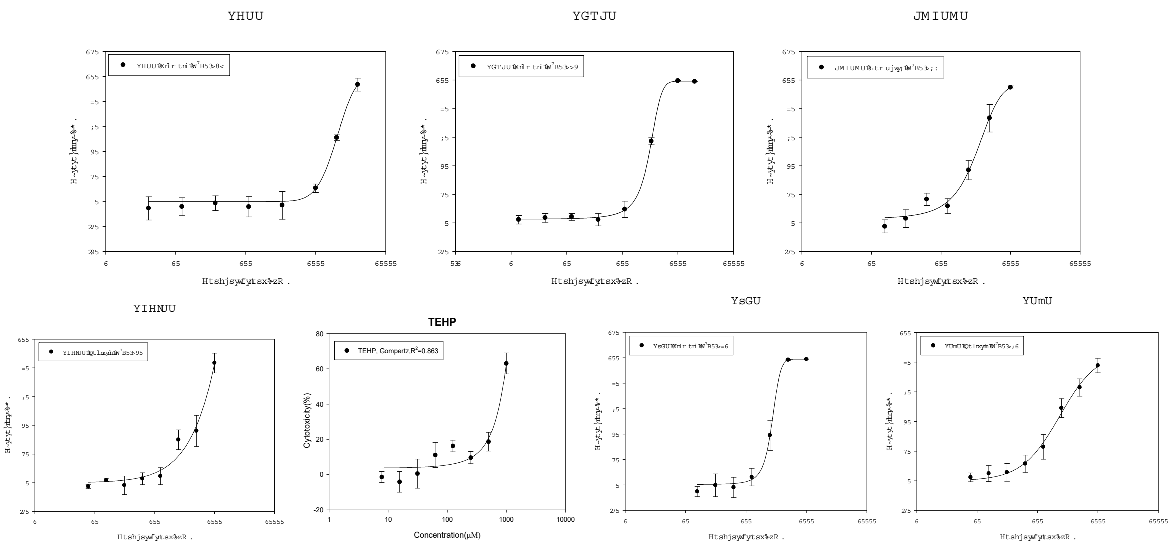

Figure S1. Dose–response curves of target single substances: (a) Environmental phenols; (b) heavy metals and their compounds; (c) per- and polyfluoroalkyl substances (PFAS); (d) organophosphate flame retardants (OPFRs). All experiments were conducted according to three independent replicates using difference cell passages. Dose–response curves were fitted using the best-fit model selection among non-linear sigmoid functions of three parameters (sigmoid, logistic, Gompertz, Weibull, Hill, and Chapman models). Fitted regression model and adjusted R squares are presented.
